# Expression of concern: Multi-walled carbon nanotubes decorated with palladium nanoparticles as a novel platform for electrocatalytic sensing applications

**DOI:** 10.1039/d4ra90114b

**Published:** 2024-09-30

**Authors:** Mehdi Baghayeri, Hojat Veisi, Hamed Veisi, Behrooz Maleki, Hassan Karimi-Maleh, Hadi Beitollahi

**Affiliations:** a Department of Chemistry, Faculty of Science, Hakim Sabzevari University P.O. Box 397 Sabzevar Iran m.baghayeri@hsu.ac.ir +98 5714003170 +98 5714003325; b Department of Chemistry, Payame Noor University 19395-4697 Tehran Iran; c Student Research Committee, Shiraz University of Medical Sciences Shiraz Iran; d Department of Chemistry, Graduate University of Advanced Technology Kerman Iran; e Environment Department, Institute of Science and High Technology and Environmental Sciences, Graduate University of Advanced Technology Kerman Iran

## Abstract

Expression of concern for ‘Multi-walled carbon nanotubes decorated with palladium nanoparticles as a novel platform for electrocatalytic sensing applications’ by Mehdi Baghayeri *et al.*, *RSC Adv.*, 2014, **4**, 49595–49604, https://doi.org/10.1039/C4RA08536A.

*RSC Advances* is publishing this expression of concern in order to alert readers that concerns have been raised over the integrity of the data published in this article. The authors have been contacted but have not responded to requests to provide raw data. An expression of concern will continue to be associated with the article until a conclusive outcome is reached.

Laura Fisher

16th September 2024

Executive Editor, *RSC Advances*

